# Risk-reducing salpingo-oophorectomy in patients with *gBRCA*-mutated breast cancer

**DOI:** 10.1007/s00404-026-08581-2

**Published:** 2026-09-28

**Authors:** Niklas Amann, Kim Wagner, Philipp Ziegler, Annika Krückel, Lena Brückner, Julia Gocke, Carolin Müller, Christoph Runz, Felix Heindl, C. Carolin Hack, Julius Emons, A. Peter Fasching, W. Matthias Beckmann, Manuel Hörner

**Affiliations:** 1 Department of Gynecology and Obstetrics, Universitätsklinikum Erlangen, Friedrich-Alexander-Universität Erlangen-Nürnberg (FAU), Comprehensive Cancer Center Erlangen-EMN (CCC ER-EMN), Erlangen, Germany; 2 Bavarian Cancer Research Center (BZKF), Universitätsstraße 21/23, 91054 Erlangen, Germany

**Keywords:** BRCA, Hereditary breast and ovarian cancer, Mastectomy, OC, RRSO

## Abstract

**Purpose:**

Breast cancer (BC) patients with pathogenic germline *BRCA1/2* mutations (*gBRCA1/2mut)* have a substantially increased risk of ovarian cancer (OC). Risk-reducing bilateral salpingo-oophorectomy (RRSO) remains the only effective preventive strategy. However, data on the utilization and time to RRSO in g*BRCA*mut BC patients are limited.

**Methods:**

This retrospective study included 148 patients with *gBRCA1/2mut* BC treated at the Hereditary Breast and Ovarian Cancer Center, University Hospital Erlangen, between 2012 and 2024. Clinical, pathological, treatment-related, and genetic data were collected and analyzed from electronic records. Time to RRSO at date of BC surgery was calculated for all patients, and stratified according guideline recommendations for RRSO, using the Kaplan–Meier method.

**Results:**

In this cohort of 148 patients with *gBRCAmut* BC, 60.8% harbored a *gBRCA1* and 39.2% a *gBRCA2* mutation. BC was diagnosed at the mean age of 47.1 years, 73.6% presenting at UICC stage I–II. 47.9% of patients presented with triple-negative BC, 38.4% of patients with hormone receptor (HR)-positive/human epidermal growth factor receptor 2 (HER2)-negative BC and 8.9% with HER2-positive BC. 101 patients (68.2%) additionally underwent RRSOs. The 12-month RRSO-free probability was 0.58 (95% CI 0.50–0.68) and the 24-month RRSO-free probability 0.39 (95% CI 0.31–0.49). Occult OC was detected in three patients at the time of RRSO during BC follow-up, four patients without RRSO were diagnosed with OC after BC.

**Conclusions:**

In real-world setting, RRSO is a common prevention in g*BRCA*mut BC follow-up. The majority of patients undergo surgery within the first two years following a BC diagnosis. Survival outcomes among patients with BC have improved substantially in recent years. Consequently, developing OC in g*BRCA*mut BC individuals still remains a risk. RRSO should therefore continue to be offered and discussed in accordance with current guidelines, even in the context of a BC diagnosis.

**Supplementary Information:**

The online version contains supplementary material available at https://doi.org/10.1007/s00404-026-08581-2.

## Introduction

Approximately one-third of all breast cancer (BC) cases are associated with a familial risk constellation [1, 2]. Hereditary breast and ovarian cancer (HBOC) can be identified in a third of patients with familial BC risk [1, 2]. So nearly, HBOC is detected in up to 10% of BC patients in total. The most frequent pathogenic variants for HBOC are located in *BRCA1* and *BRCA2* genes [3]. In addition to an increased BC risk in up to 70%, mutation carriers have also a lifetime ovarian cancer (OC) risk ranging from 17 to 49% [4, 5].

International consortia such as CIMBA have enabled the development of cross-national recommendations for cancer surveillance and risk reduction. Structured surveillance programs are now potentially established in several countries, including the United States, the United Kingdom, and Germany [6]. In Germany, the German Consortium for Hereditary Breast and Ovarian Cancer (GC-HBOC) has implemented a nationwide, standardized surveillance program [7, 8]. Academic oncology centers are collaborating and forming a knowledge-generating network. With such structured, quality-assured care survival outcomes are significantly improved [7, 9–11].

Whereas BC surveillance strategies, such as annual breast magnetic resonance imaging (MRI), have been integrated into routine care, no standardized surveillance exists in OC screening. National recommendations remain heterogeneous [12, 13]. The European Society for Medical Oncology recommends semiannual gynaecologic ultrasound while acknowledging limited evidence, whereas the GC-HBOC does not support routine adnexal ultrasound [14].

Currently, the only effective option for OC prevention is adnexectomy [15]. In 2002, Kauff et al. showed a significant risk reduction of BC and OC in patients undergoing risk-reducing salpingo-oophorectomy (RRSO) (HR 0.25; 95% CI 0.08–0.74)[16]. RRSO was also associated with a significant risk reduction in OC in g*BRCA*mut patients in a meta-analysis of Rebbeck et al. (HR 0.21; 95% CI 0.12–0.39) [17].

In a prospective multi-center cohort study of 2482 patients with *BRCA1* or *BRCA2* mutations, women who underwent RRSO had a lower risk of OC, with a HR of 0.14 (95% CI, 0.04–0.59) in the subgroup of patients with prior BC [18]. Undergoing RRSO was associated with lower all-cause mortality in patients with prior BC (HR 0.30; 95% CI, 0.17–0.52) and also lower OC-specific cancer mortality (HR 0.1; 95% CI 0.01–1.42) [18]. Furthermore, in 2025, Blondeaux et al. demonstrated a significantly better overall survival (OS) in g*BRCA*mut BC patients undergoing a RRSO (HR 0.58; 95% CI 0.48–0.71) [19].

Without any RRSO, another prospective cohort study for patients with g*BRCA1*mut BC, the cumulative risk of OC was 14.9% in 15-year BC follow-up, respectively 5.1% for *BRCA2* carriers [20]. Further data showed a 10-year risk for OC after stage I–II g*BRCA*mut BC in 12.7% for *BRCA1* and 6.8% for *BRCA2* [21]. These results still support a recommendation for RRSO in g*BRCA*mut BC patients. In addition, patients appear to be less reluctant to undergo RRSO [22, 23].

In patients with g*BRCA*mut BC, the time to RRSO remains a frequent topic of discussion during genetic counseling and routine clinical care. However, high-quality evidence to inform this decision is lacking. Consequently, whether RRSO is performed immediately after completion of BC treatment or deferred until later during follow-up remains uncertain.

Previous studies, including those by Blondeaux et al. and Willert et al., have demonstrated that RRSO is associated with a significant reduction in OC incidence, while the uptake of risk-reducing surgery has increased substantially over recent years [19, 24]. Nevertheless, evidence defining the time to RRSO remains limited. Current national and international guidelines recommend offering RRSO from the age of 35 years in *BRCA1* and 40 years in *BRCA2* pathogenic variant carriers [25]. However, they do not provide specific recommendations regarding the timing of surgery in relation to BC treatment.

The primary objective of this study was to evaluate the RRSO-free probability in patients with g*BRCA*mut BC to determine the interval between BC operation and RRSO in a real-world setting. In addition, this study aims to characterize the patient cohorts and assess the incidence OC in RSSO versus the occurrence of OC in non-operated g*BRCA*mut BC patients during follow-up.

## Methods

### Study site

Between January 1, 2012 and December 31, 2024, 3,599 individuals presented at the HBOC Center at the University Hospital Erlangen. In order to qualify for genetic counseling related to HBOC, patients had to fulfill the testing criteria established by the German Cancer Society. Patient data were collected retrospectively and documented in an electronic case report.

For patients with BC, genetic testing is recommended if BC was diagnosed before the age of 36 years, if triple-negative breast cancer (TNBC) was diagnosed before the pre-specified cut-off age, which changed over the recruitment period, or if ovarian/peritoneal/fallopian tube cancer was diagnosed before the age of 80 years, even in the absence of a suspicious family history. These criteria are reviewed annually and adapted based on the latest scientific evidence, resulting in changes to the testing guidelines during the study period. As additional inclusion criteria are incorporated over time, the study cohort is expected to increase substantially over the observation period. Following the introduction of poly(ADP-ribose) polymerase inhibitors (PARPi) for the locally advanced OC and human epidermal growth factor receptor 2 (HER2)-negative BC associated with pathogenic *BRCA1/2* mutations, germline testing also gained therapeutic relevance irrespective of family history. Testing can be initiated with a therapeutic indication without fulfilling the testing criteria.

### Patients

At the time of data cut-off (December 31, 2024), 3,599 patients were registered in the database. Of these, non-affected individuals (*n* = 1,050), affected patients with non-BC (*n* = 383), and patients without a *gBRCA* mutation (*n* = 1,954) were excluded. A g*BRCA* mutation was detected in 212 patients with BC/DCIS. From this population, data were unavailable for an additional 61 patients, mainly treated in other certified BC centers. As HBOC testing is restricted to selected centers, these patients presented in this clinic just for genetic counseling and testing. 3 male patients were excluded furthermore. Ultimately, 148 patients with g*BRCAmut* BC/DCIS were included in this descriptive analysis. Follow-up assessments for the BC setting are updated every 6 months until 5 years after BC diagnosis. An intensified breast cancer surveillance (IBCS) is recommended for g*BRCA*mut BC patients without a mastectomy.

**Fig. 1:**
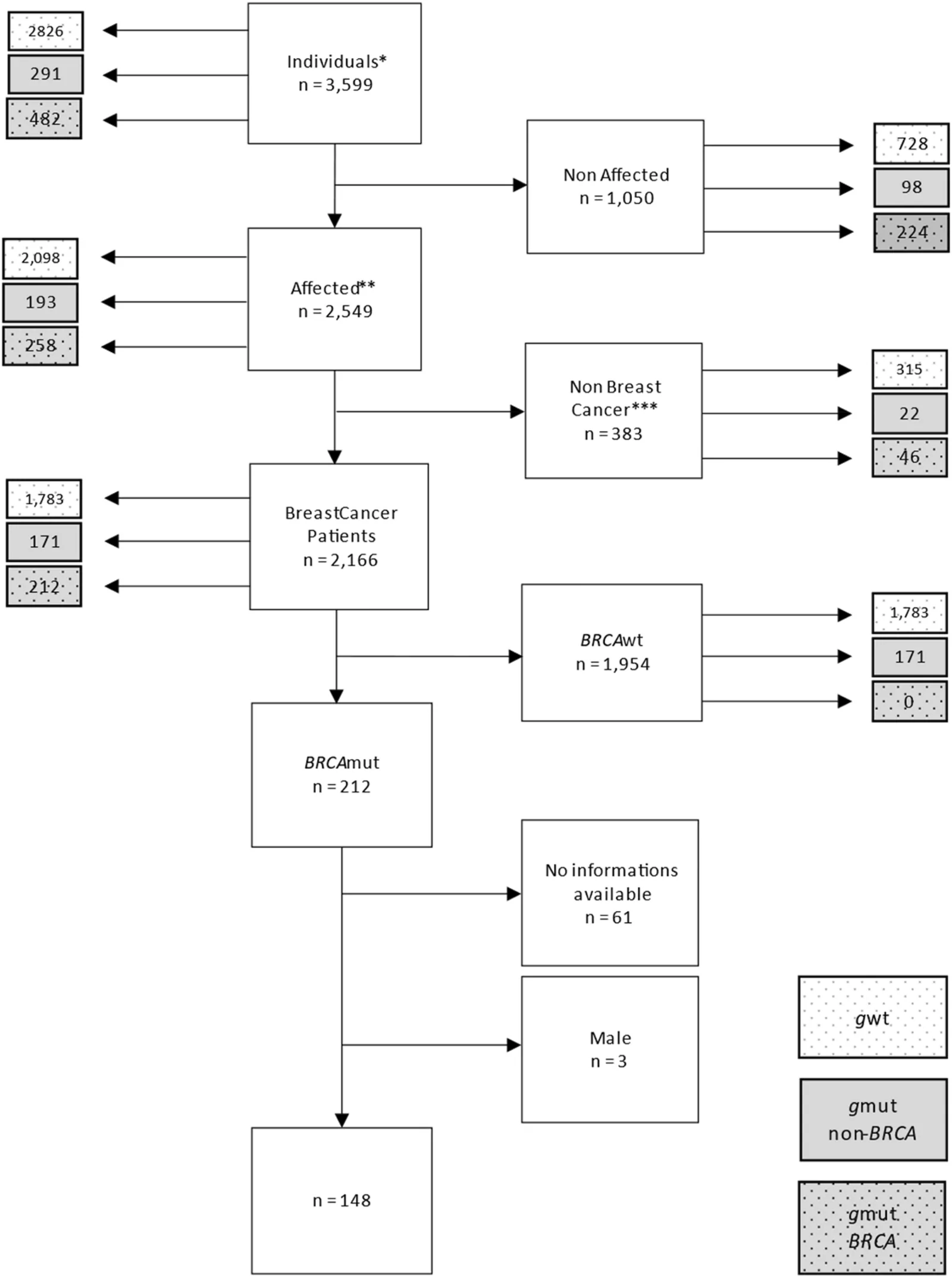
Patient selection for *gBRCAmut* BC/DCIS out of 3,599 patients tested for HBOC at University Hospital Erlangen. * = positive HBOC criteria (preventive, diagnostic, therapeutic testing), ** = Diagnosed *BC* Borderline-tumors, STIC, OC, Peritoneal Cancer, Cancer of the Fallopian Tube, *** = Borderline-Tumors, STIC, OC, Peritoneal Cancer, Cancer of the Fallopian Tube

Epidemiological, clinicopathological, and treatment-related data regarding the cancer diagnosis and treatment were assessed. All specimens obtained from patients who underwent RRSO at our center were processed and histopathologically examined according to the internationally accepted Sectioning and Extensively Examining the Fimbriated End (SEE-FIM) protocol. In addition, information on germline mutation analysis and subsequent management, specifically participation in IBCS programs or the performance of RRSO, was collected and evaluated. The patient flow chart is shown in Fig. 1.

### Structured process for genetic testing

Patients eligible for genetic counseling are identified through a systematic assessment of the German Cancer Society’s inclusion criteria. If the criteria are met, patients can undergo counseling at the HBOC center. The counseling is provided by trained medical personnel who have completed the GC-HBOC basic module and hold additional qualifications in specialized human genetics counseling.

Upon completion of the counseling session and after obtaining written informed consent, genetic testing is initiated. A blood sample is collected and analyzed using next-generation sequencing technology. The specific methods employed are detailed in the molecular genetic report, which also includes information about the genes analyzed and any identified genetic alterations. More than one genetic variant is possible. Genetic variants are classified according to the current Human Genome Variation Society nomenclature using the American College of Medical Genetics and Genomics (ACMG) classes.

Once the molecular genetic results are available, findings are discussed with the patient. In cases where a pathogenic or likely pathogenic variant is detected (classified as ACMG class 4 or 5), risk-reducing surgical options (e.g., mastectomy, adnexectomy) or enrollment in an IBCS program are considered depending on the specific gene mutation. If the variant is of uncertain significance (ACMG class 3), patients are advised to return for re-evaluation of the variant after three years. No immediate therapeutic or diagnostic measures are taken in such cases. A negative (non-pathogenic) result has no clinical implications. It should be emphasized that certain patient groups who meet the criteria for genetic testing may remain eligible for additional interventions even if their test results are negative or of uncertain significance. For instance, in Germany, patients with BC diagnosed before the age of 36 years may still participate in the IBCS program until they reach the age of 50 years, irrespective of genetic findings.

### Data collection

Patient data for the period from January 1, 2012 to December 31, 2024, at the HBOC Center at the University Hospital Erlangen were collected retrospectively by trained personnel. Medical histories and results of human genetic testing were documented in an electronic case report form using Microsoft Access 365 (Microsoft, Redmond, WA, USA). Patient and tumor characteristics were described using summary statistics. Frequency and percentage were used for categorical variables. Plausibility checks issued regarding the research question, and on-site monitoring was performed.

### RRSO-free probability analysis

#### Exclusions

For Kaplan–Meier analysis, regarding RRSO-free probability of all 148 patients, 21 patients were excluded due to missing time to RRSO in months. Subsequently, 2 patients had to be excluded due to non-positive time to RRSO. For all patients included in the RRSO-free probability analysis, germline testing was performed in the context of the BC diagnosis within a time window of approximately ±3 months relative to BC surgery. In patients who received neoadjuvant chemotherapy, germline testing performed up to 6 months before BC surgery was considered eligible. Patients with the diagnosis of HBOC outside this time window relative to BC surgery were thus excluded in the time to RRSO analysis. Time to RRSO was right-censored at 240 months (4 patients). Additionally, patients were excluded if germline testing was performed after the diagnosis of advanced or metastatic BC for therapeutic decision-making (11 patients). Patients were also excluded if they already underwent salpingo-oophorectomy due to metachronous OC (1 patient), if no further clinical information was available because surgery had been performed at an external institution (4 patients), or if the BC surgery was close to the end of follow-up (< 2months; 1 patient). Finally, 125 patients were eligible for the RRSO-free probability analysis.

#### Outcome variable

RRSO-free probability was defined from the date of BC operation to the date of RRSO or the last RRSO-free date known. The RRSO-free last date known was the date of death if death happened without operation. If a patient was lost to follow-up before death or operation, the last RRSO-free date was defined as the date of the last follow-up. Otherwise, the last known RRSO-free date was the date of data extraction, December 31st, 2024. RRSO-free probability was right-censored at 240 months (= 20 years).

#### Subgroups (RRSO recommendation)

For the collective, it was of interest whether there was a difference in RRSO-free probability with respect to the guideline recommendations for RRSO in patients with a g*BRCA* mutation, which was known at the date of BC operation:

A. Patients with a g*BRCA1* mutation and age 35 years or older or patients with a g*BRCA2* mutation and age 40 years or older
B. Patients with a g*BRCA1* mutation and age less than 35 years or patients with a g*BRCA2* mutation and age less than 40 years

For patients in group A, RRSO was recommended; for patients in group B, it was not (yet) recommended.

#### Analysis

RRSO-free probability with 95% confidence intervals (CIs) and median time to RRSO were estimated using the Kaplan–Meier product limit method. The 95% CI of the RRSO-free probability was computed using the method of Brookmeyer and Crowley [26]. Calculations were performed with R statistical computing (version 4.5.1) within the RStudio environment.

## Results

### Patient characteristics

The final study cohort for descriptive analysis includes 148 patients with g*BRCA*mut BC. In 90 patients (60.8%), g*BRCA1* mutation was detected; in 58 patients, g*BRCA2* mutation (Supplementary Table 1). In Table 1, demographic and baseline tumor characteristics are shown. The mean age at diagnosis was 47.1 years. 20 patients (13.5%) were aged <35 years at the time of diagnosis. A total of 47.9% of patients were diagnosed aged 35–49 years, 31.1% aged 50–69 years, and 9 patients (6.1%) were aged ≥70 years. 58.4% patients opting for RRSO were aged 35–49 years, 8 patients (7.9%) were aged <35 years at the time of diagnosis (Table 2).

**Table 1:** Patient and tumor characteristics with and without RRSO, showing mean and standard deviation or frequency and percentage

	All Patients (*N* = 148)	Patients with RRSO (*n* = 101)	Patients without RRSO (*n* = 47)
Age (years) (*n*/%)			
Mean (SD)	47.1 (± 12.4)	46.7 (± 8.2)	48.2 (± 16.2)
Median (range)	44.5 (21–82)	44.5 (29–72)	45 (21–82)
<35	20 (13.5)	8 (7.9)	12 (25.5)
≥35, <50	71 (47.9)	59 (58.4)	12 (25.5)
≥50, <70	46 (31.1)	31 (30.7)	15 (31.9)
≥70	9 (6.1)	3 (3.0)	6 (12.8)
No information available	2 (1.4)	0	2 (4.3)
*BRCA* Mutation (*n*/%)			
*BRCA1*	90 (60.8)	61 (60.4)	29 (61.7)
*BRCA2*	58 (39.2)	40 (39.6)	18 (38.3)
Additional HGSC (n/%)			
No	138 (93.2)	98 (97.1)	40 (85.1)
Yes	10 (6.8)	3 (2.9)	7 (14.9)
• Before BC	2 (20.0)	0	2 (28.6)
• Synchronous	1 (10.0)	0	1 (14.2)
• After BC	7 (70.0)	3 (100)	4 (57.2)
BC Relapse (n/%)			
No	112 (75.6)	81 (80.2)	31 (66.0)
De novo mBC	9 (6.1)	4 (4.0)	5 (10.6)
Yes	27 (18.2)	16 (15.8)	11 (23.4)
• Local	11 (40.7)	7 (43.8)	4 (36.3)
• Local + Advanced	3 (11.1)	1 (6.2)	2 (18.2)
• Advanced	13 (48.1)	8 (50.0)	5 (45.5)
Time to relapse (months) (*n*/%)			
≤18	3 (11.1)	1 (6.3)	2 (18.2)
>18, ≤36	9 (33.3)	4 (25.0)	6 (54.5)
>36, ≤60	7 (26.0)	5 (31.2)	1 (9.1)
>60	8 (29.6)	6 (37.5)	2 (18.2)
UICC Stage (*n*/%)			
0 (DCIS)	2 (1.4)	1 (1.0)	1 (2.2)
I	44 (29.7)	35 (34.6)	9 (19.1)
II	65 (43.9)	45 (44.5)	20 (42.5)
III	8 (5.4)	4 (4.0)	4 (8.5)
IV	8 (5.4)	2 (2.0)	6 (12.8)
No information available	21 (14.2)	14 (13.9)	7 (14.9)
BC subtype (*n*/%) *			
HR pos	56 (38.4)	36 (36.0)	20 (43.5)
HER2 pos	13 (8.9)	10 (10.0)	3 (6.5)
TNBC	70 (47.9)	52 (52.0)	18 (39.1)
No information	7 (4.8)	2 (2.0)	5 (10.9)

^*^ = DCIS (2 cases) is excluded out of BC subtypes

**Table 2:** BC risk-reducing operations in *gBRCA*mut BC patients with and without RRSO

	All Patients (*N* = 148)	Patients with RRSO (*n* = 101)	Patients without RRSO (*n* = 47)
Breast operation (*n*/%)			
Breast-conserving therapy	37 (25.0)	25 (24.8)	12 (25.5)
Mastectomy	98 (66.2)	73 (72.3)	25 (53.2)
No information available	13 (8.8)	3 (2.9)	10 (21.3)
Reconstruction (*n*/%)			
Total	98	73	25
Subcutaneous implants	21 (21.4)	15 (20.5)	6 (24.0)
Sub-pectoral implants	31 (31.6)	23 (31.5)	8 (21.0)
No implants	46 (47.0)	35 (48.0)	11 (55.0)
RRSO (*n*/%)			
Yes	101 (68.2)	−	−
No	47 (31.8)	−	−
Time to RRSO (months) (*n*/%)			
≤12	−	52 (51.5)	−
>12—≤24	−	23 (22.8)	−
>24—≤60	−	9 (8.9)	−
>60	−	16 (15.8)	−
Before BC	−	1 (1.0)	−

10 patients were additionally diagnosed with HGSC (Table 1). 7 patients were diagnosed with HGSC during BC follow-up (Tables 1, 3, 4). By December 2024, 27 patients (18.2%) had experienced a BC relapse. 3 patients recurred with BC within 18 months, and another 9 within 36 months.

In 73.6% of patients, the UICC stage at initial BC diagnosis was stage I or II (Table 1). 8 patients (5.4%) were diagnosed at UICC stage III and IV, respectively. 56 patients (38.4%) had hormone receptor (HR)-positive, HER2-negative disease, 13 (8.9%) patients HER2-positive BC and 70 patients (47.9%) TNBC (Table 1). g*BRCA1* mutation was detected in 67.2% (with RRSO)/55.2% (without RRSO) in TNBC patients, g*BRCA2* mutation in 56.4% (with RRSO)/70.6% (without RRSO) in HR-positive, HER2-negative BC patients (Supplementary Tables 2, 3).

### Surgical management for gBRCA1/2mut BC patients

A total of 37 patients (25.0%) underwent breast-conserving therapy (Table 2). 98 patients (66.2%) underwent mastectomy. 46 patients opted for no further reconstruction, 52 patients for a reconstruction with either subcutaneous or sub-pectoral implants. Patients who opted for RRSO also underwent mastectomy in 72.3% of cases, while patients who did not opt for RRSO underwent mastectomy in 53.2% of cases.

A total of 101 patients received RRSO. Of those patients who received RRSO, 49.2% of the patients with g*BRCA1*mut BC and 55.0% with g*BRCA2*mut BC underwent RRSO within the first 12 months (Supplementary Tables 4, 5).

### OC for patients with gBRCAmut BC

Three out of 101 patients with *gBRCAmut* BC undergoing RRSO in follow-up were diagnosed with occult HGSOC (Table 3). Patients 2 and 3 received the RRSO seven months after BC operation. Patient 1 underwent RRSO more than 15 years. All of the patients received a laparotomy as operative OC treatment. One was diagnosed with a FIGO IIIA stage; for the other two patients, tumor stages were FIGO IA and IC. No BC or OC relapse was reported until data cut (December 2024). Patients 2 and 3 were treated with a taxane-based neoadjuvant medical therapy for BC and both underwent bilateral skin-sparing mastectomy (Table 3). Due to the short interval since completion of BC chemotherapy, no decision was made to initiate further OC chemotherapy. As Patient 2 was diagnosed with OC stage IIIA, poly(ADP-ribose) polymerase inhibitor (PARPi) treatment was started after OC operation.

Seven out of 47 patients not undergoing RRSO developed OC either before (N=2), synchronous (N=1), or after (N=4) BC diagnosis (Table 1). BC was diagnosed between the age of 38 and 65 years (Table 4). UICC BC stage was stage IA, three patients underwent breast-conserving therapy, one patient had a mastectomy. One patient locally recurred with BC after 34 months. During BC follow-up, OC was initially diagnosed after 91 to 449 months. Three patients underwent laparotomy with a following adjuvant platin-based chemotherapy. One patient only received palliative systemic treatment (FIGO IVA) (Table 4).

**Table 3:** *gBRCA*mut BC patients with occult OC diagnosis in RRSO

Patients	Patient 1	Patient 2	Patient 3
Germline mutation	*BRCA1*	*BRCA1*	*BRCA2*
Age at BC diagnosis (years)	31	47	71
UICC	IIA	IIB	IA
BC subtype	TNBC	HR pos	TNBC
BC operation	Breast-conserving therapy	Bilateral SSM	Bilateral SSM
BC medical treatment	Neoadjuvant chemotherapy (Epirubicin+Cyclophosphamide, Docetaxel)	Neoadjuvant chemotherapy in GeparOla-Trial (Paclitaxel+Olaparib, Epirubicin+Cyclophosphamide), Endocrine Treatment (Letrozole)	Neoadjuvant chemotherapy (Carboplatin+Paclitaxel)
BC relapse (months)	No	No	No
Age at OC diagnosis (years)	48	48	72
Time to RRSO (months)	184	7	7
FIGO	IC	IIIA	IA
OC subtype	HGSOC	HGSOC	HGSOC
OC operation	Laparotomy	Laparotomy	Laparotomy
Medical treatment	Adjuvant chemotherapy (Carboplatin+Paclitaxel)	PARPi	-

**Table 4:** *gBRCA*mut BC patients with OC diagnosis without RRSO during follow-up care

Patients	Patient 1	Patient 2	Patient 3	Patient 4	Patient 5
Germline mutation	*BRCA1*	*BRCA2*	*BRCA2*	*BRCA1*	*BRCA2*
Age at BC diagnosis (years)	65	38	53	57	61
UICC	IIA	Unknown	IA	IA	IA
BC subtype	HR-positive	Unknown	HR-positive	Unknown	TNBC
BC operation	Mastectomy	Mastectomy	Breast-conserving therapy	Breast-conserving therapy	Breast-conserving therapy
BC medical treatment	Endocrine treatment (Letrozole)	Unknown	Endocrine treatment (Tamoxifen)	Adjuvant therapy	Adjuvant therapy (Epirubicin/ Cyclophosphamide/ Trastuzumab)
BC Relapse (months)	No	No	34	No	No
Age at OC diagnosis (years)	65	74	64	81	69
Time to OC diagnosis (months)	0	449	148	298	91
FIGO	IIIC	IIIC	IIIC	IVA	IA
OC subtype	HGSOC	HGSOC	HGSOC	HSGOC	HGSOC
OC operation	Laparotomy	Laparotomy	Laparotomy	-	Laparotomy
Medical treatment	Adjuvant chemotherapy (Carboplatin/ Paclitaxel/ Bevacizumab)	Adjuvant chemotherapy (Carboplatin/ Paclitaxel/ Bevacizumab)	Adjuvant chemotherapy (Carboplatin/ Paclitaxel/ Bevacizumab)	Palliative chemotherapy (Carboplatin/ Paclitaxel/ Bevacizumab)	Adjuvant chemotherapy (Carboplatin)

### RRSO-free probability analysis

**Fig. 2:**
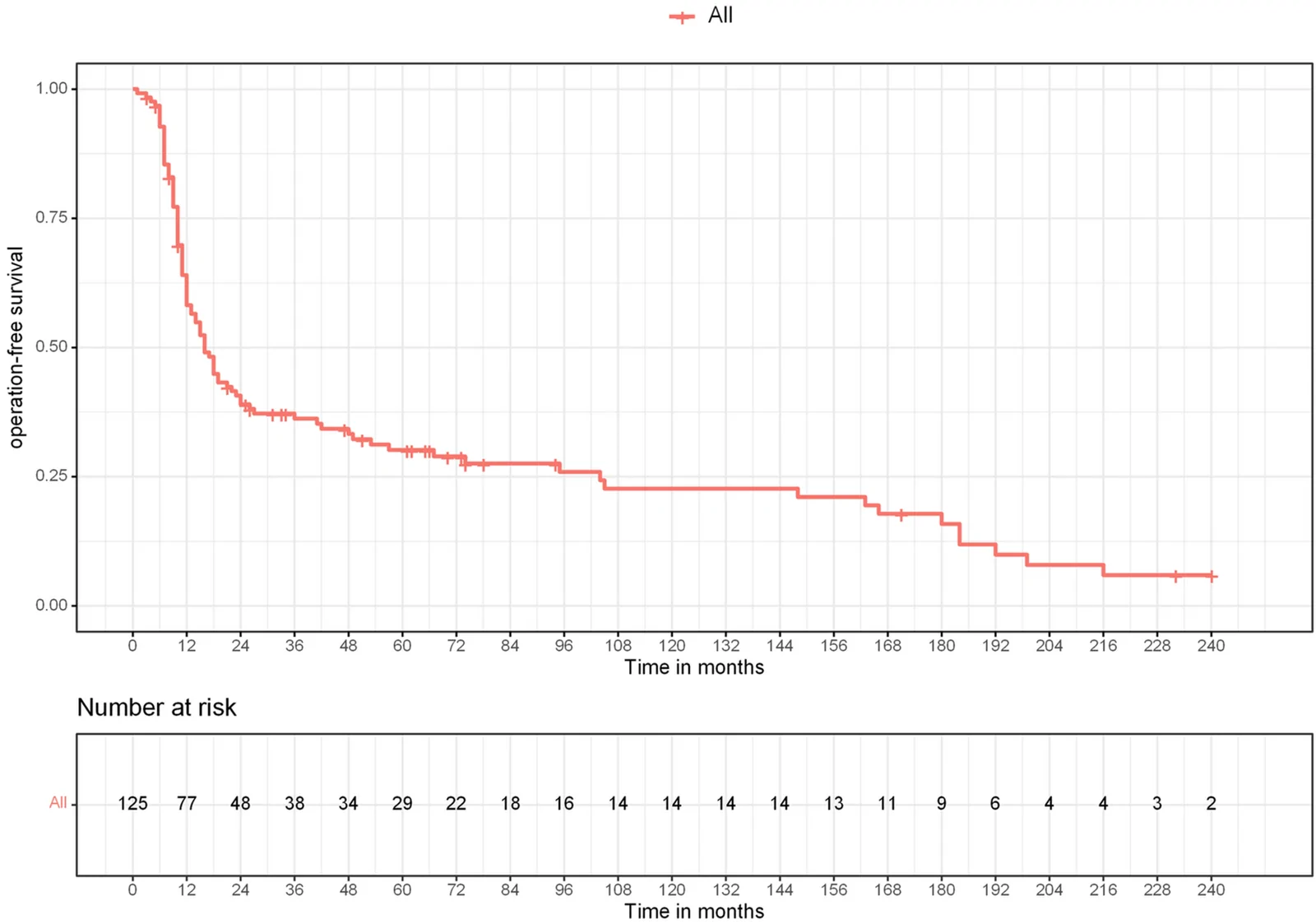
Time to RRSO at date of BC surgery (in months) for *gBRCA1/2mut* patients

For the overall population analysis, the median time to RRSO was 16.0 months (95% CI 13.0–24.0), the Kaplan–Meier graph is shown in Fig. 2. The RRSO-free probability at 6 months was 0.93 (95% confidence interval [CI] 0.88–0.97), at 12 months 0.58 (95% CI 0.50–0.68) and 0.39 (95% CI 0.31–0.49) at 24 months (Supplementary Table 6).

**Fig. 3:**
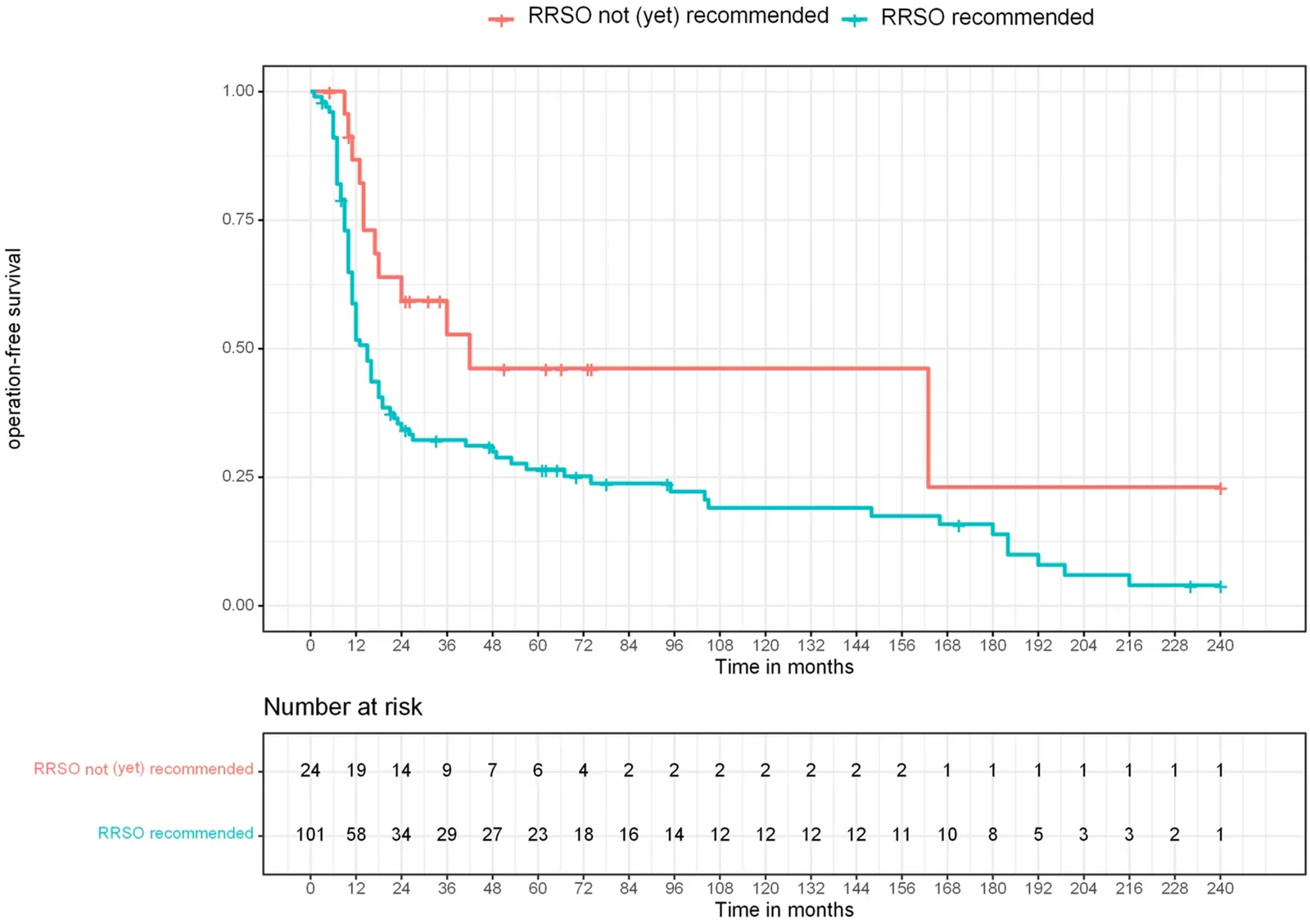
Time to RRSO with respect to guideline recommendations for RRSO at date of BC surgery (in months) for *gBRCA1/2mut* patients. RRSO was recommended for patients with a *gBRCA1* mutation and age 35 years or older or patients with a *gBRCA2* mutation and age 40 years or older

For patients with a recommendation for RRSO (Supplementary Table 7), the RRSO-free probability at 6 months was 0.91 (95% CI 0.86–0.97), at 12 months 0.52 (95% CI 0.43–0.63) and 0.34 (95% CI 0.26–0.45) at 24 months. For patients for which RRSO was not (yet) recommended, the RRSO-free probability at 6 months was 1.00 (95% CI 1.00–1.00), at 12 months 0.87 (95% CI 0.74–1.00) and 0.59 (95% CI 0.42–0.84) at 24 months (Supplementary Table 7) Fig. 3.

## Discussion

In the single-center retrospective analysis of patients with g*BRCA*mut BC, most patients underwent RRSO. Occult OC was detected in three out of 101 patients due to RRSO. The median time to RRSO was 16.0 months.

For early detection and risk reduction of OC, transvaginal ultrasonography may be considered in addition to RRSO [14]. However, its clinical benefit remains controversial. An effect on OS has yet to be clearly established [27]. Nonetheless, available data suggest that malignancy could be detected at an earlier stage, which may in turn allow for less extensive surgical intervention [28]. This could result in higher rates of complete resection and, consequently, improved postoperative outcomes. In Germany, however, a more cautious stance is adopted regarding OC screening by ultrasonography. RRSO remains the most effective strategy for risk reduction and continues to be regarded as the standard of care [29]. Current guidelines advise that RRSO be performed between 35 and 40 years of age in *BRCA1* carriers and between 40 and 45 years in *BRCA2* carriers. The influence on BC still remains uncertain [18, 30]. In addition, the impact of RRSO on contralateral breast cancer (CBC) risk in g*BRCA1/2* mutation carriers remains controversial. While some studies have demonstrated a reduction in CBC risk following RRSO, this protective effect has not been consistently confirmed in subsequent analyses [18, 31–33].

Two-thirds of patients underwent RRSO. For the overall population analysis, the 12-month RRSO-free probability was 0.58 (0.50, 0.68) and 0.39 (0.31, 0.49) for 24-month RRSO-free probability. Especially in patients with BC before the age of 35 years, RRSO was performed later but still within the internationally recommended timeframe for risk-reducing surgery [25]. There is no recommendation for RRSO before the age of 35 years, except for 5 years before the youngest OC age.

Premenopausal women experience immediate menopause, which may substantially affect quality of life and sexual well-being [29]. Potential adverse effects on bone health and cardiovascular risk have been described although large registry analyses have not consistently demonstrated an increased cardiovascular risk [34]. As an alternative in future, bilateral salpingectomy with delayed oophorectomy may be offered to younger patients seeking maximal risk reduction while avoiding premature menopause. Salpingectomy alone may already confer a reduction in ovarian cancer risk [6, 34]. This approach is currently being investigated in g*BRCA1/2* mutation carriers in the TUBA-WISP II trial, with results pending [35].

A standardized pathological examination of RRSO specimens according to the SEE-FIM protocol to avoid missing occult carcinomas is important [6]. Occult carcinomas are detected in approximately 1–5% of cases at the time of RRSO [29, 36–40]. Finch et al. identified 46 invasive carcinomas among 1,390 g*BRCA1/2* mutation carriers, corresponding to a prevalence of 3% [29]. In our cohort, occult ovarian carcinoma was likewise identified in three out of 101 patients undergoing RRSO: two cases among 61 g*BRCA1* mutation carriers and one case among 40 *gBRCA2* mutation carriers.

In the context of precision oncology, OS in BC patients is improving. It may therefore be assumed that the risk of second primary malignancies could also increase in g*BRCA*mut patients during BC follow-up. Apostol et al. have demonstrated an ongoing OC in g*BRCA*mut BC patients, with a pronounced high risk in g*BRCA1* mutations carriers [20]. Accordingly, RRSO should continue to be recommended for g*BRCA*mut BC patients as a preventive strategy. In contrast, in g*BRCA*mut OC patients, risk-reducing breast surgery should be considered with caution. Recent data indicate an overall very low BC incidence within this specific patient population [41].

In this analysis, real-world data of median time to RRSO after BC surgery in g*BRCA*mut BC patients are presented for the first time. This is an important issue in counseling of affected patients. Early implementation appears to be the preferred approach for the majority of patients. However, it should be noted that the generalizability and translation of these findings into routine clinical practice remain limited.

Our study has several limitations. The retrospective design leads to the introduction of several biases; selection bias cannot be excluded as patients who underwent RRSO may differ systematically from those who did not. Missing or incomplete data, differing follow-up times and other confounding factors may have affected the outcome assessment. Ascertainment bias likely resulted in underreporting in our real-world trial similar to other real-world analyses, for example, when patients opted to undergo RRSO elsewhere. Owing to the single-center design, the cohort size is limited. The relatively small cohort size precluded meaningful inferential statistical analyses. Therefore, the data were predominantly analyzed descriptively. Given the limited sample size, the risk of statistical error was considered substantial. In addition, the single-center design may have introduced selection bias related to local clinical counseling and referral practices. Nevertheless, genetic counseling and clinical decision-making were conducted in accordance with current national and international guidelines. A further limitation is that data were collected in Germany only. Here, transvaginal ultrasonography for early detection is not recommended [42]. Only risk-reducing surgery is reimbursed by health insurance providers. Owing to the limited evidence base, no guideline recommendation for transvaginal ultrasonography currently exists in Germany in contrast to the situation in some other countries. This more directive approach to genetic counseling should be taken into account when interpreting the results. Further retrospective and prospective studies are warranted to better assess outcomes, especially for time to RRSO and survival analysis.

## Conclusion

The time to RRSO in g*BRCA1/2* mutation carriers remains challenging. The procedure is generally recommended between the age of 35 and 45 years, depending on the specific mutation. When HBOC is identified due to BC diagnosis, the 12-month RRSO-free probability was 0.58. The median time to RRSO was 16.0 months. The rate of occult carcinomas in RRSO appears to be low. Nevertheless, given the continuous improvements in BC treatment and the associated increase in OS, the cumulative risk of subsequent malignancies remains clinically relevant over time. Despite a prior BC diagnosis, RRSO should continue to be discussed with g*BRCA*mut patients during BC follow-up. These data may provide valuable support during this complex decision-making process and facilitate shared decision-making to determine the most appropriate, individualized timing of RRSO.

## Supplementary Information

Below is the link to the electronic supplementary material.

Supplementary file1 (DOCX 65 KB)

## Data Availability

No datasets were generated or analyzed during the current study.

## Abbreviations

ACMG: American College of Medical Genetics and Genomics
BC: Breast cancer
*BRCA*: Breast cancer gene
*gBRCAmut*: Germline *BRCA*-mutated
*BRCAwt*: *BRCA*-Wild typ
CI: Confidence interval
CRC: Contralateral breast cancer
FIGO: Fédération Internationale de Gynécologie et d'Obstétrique
GC-HBOC: German Consortium for Hereditary Breast and Ovarian Cancer (DK-FBREK)
HBOC: Hereditary breast and ovarian cancer
HER2: Human Epidermal Growth Factor Receptor 2
HGSC: High-grade serous carcinoma of the ovary, peritoneum or fallopian tube
HR: Hormone receptor
IBCS: Intensified breast cancer surveillance
mBC: Metastatic breast cancer
MRI: Magnetic resonance imaging
MRM: Modified radical mastectomy
OC: Ovarian cancer
OS: Overall survival
PARPi: Poly(ADP-ribose) polymerase inhibitor
RRBM: Risk-reducing bilateral mastectomy
RRSO: Risk-reducing salpingo-oophorectomy
SEE-FIM: Sectioning and Extensively Examining the Fimbriated End
SSM: Skin-sparing mastectomy
STIC: Serous tubal intraepithelial carcinoma
TNBC: Triple-negative breast cancer
UICC: Union Internationale Contre le cancer
