# Supplementary material for: Risk-reducing salpingo-oophorectomy in patients with gBRCA-mutated breast cancer
Source: Arch Gynecol Obstet. 2026 Sep 28;313(1):278. doi: 10.1007/s00404-026-08581-2 (PMC13619706; doi:10.1007/s00404-026-08581-2)
Supplement: Supplementary file 1 [file 404_2026_8581_MOESM1_ESM.docx]

**Supplementary Table 1** Coding change in *gBRCA*mut patients with breast cancer.

|  | BRCA Mutation | Coding Change |
| --- | --- | --- |
| 1 | BRCA1 | c.5266dupC |
| 2 | BRCA1 | c.4689C>G |
| 3 | BRCA1 | c.5096G>A |
| 4 | BRCA1 | c.181T>G |
| 5 | BRCA1 | c.4533_4534del |
| 6 | BRCA1 | c.181 T>G heterozygot |
| 7 | BRCA1 | c.3481_3491del11 |
| 8 | BRCA1 | c.1504_1508delTTAAA |
| 9 | BRCA1 | c.329dupA |
| 10 | BRCA1 | c.3481_3491del11 |
| 11 | BRCA1 | c.(507 4+1_5075-1)_(5193+1_5194-l)del |
| 12 | BRCA1 | c.798_799delTT heterozygot |
| 13 | BRCA1 | c.5266dupC |
| 14 | BRCA1 | c.66dupA |
| 15 | BRCA1 | c.5266dup |
| 16 | BRCA1 | c.5266dupC |
| 17 | BRCA1 | c.5266dupC |
| 18 | BRCA1 | c.5266dup |
| 19 | BRCA1 | c.5406+4A>G heterozygot |
| 20 | BRCA1 | c.66dup |
| 21 | BRCA1 | c.135-2A>G |
| 22 | BRCA1 | c.2551G> T |
| 23 | BRCA1 | c.343_344del |
| 24 | BRCA1 | c.3481_3491del11 |
| 25 | BRCA1 | c.3481_3491del11 |
| 26 | BRCA1 | c.3481 3491del11 |
| 27 | BRCA1 | c.5266dupC |
| 28 | BRCA1 | c.5266dupC |
| 29 | BRCA1 | c.5153-2delA, heterozygot |
| 30 | BRCA1 | c.5266dupC |
| 31 | BRCA1 | c.68 69del heterozygot |
| 32 | BRCA1 | c.302-2A>C |
| 33 | BRCA1 | c. 798_799del |
| 34 | BRCA1 | c.3481_3491del |
| 35 | BRCA1 | c.1687C>T |
| 36 | BRCA1 | c.181T>G heterozygot |
| 37 | BRCA1 | c.(?_-200)_(441+1_442-1)del |
| 38 | BRCA1 | c.1793T>A |
| 39 | BRCA1 | c.2411_2412del |
| 40 | BRCA1 | c.3481_3491del |
| 41 | BRCA1 | c.3018_3021del |
| 42 | BRCA1 | c.2411_2412delAG |
| 43 | BRCA1 | c.3756_3759del heterozygot |
| 44 | BRCA1 | g.(? 41256139) (41276132 ?)del, heterozygot |
| 45 | BRCA1 | c.1261G>T |
| 46 | BRCA1 | c.329dup |
| 47 | BRCA1 | c.(? _-19-1)_(441 +1_ 442-1)del |
| 48 | BRCA1 | c.2411_2412del |
| 49 | BRCA1 | c.(?_-200)_(80+1_81-l)del |
| 50 | BRCA1 | c.5266dup heterozygot |
| 51 | BRCA1 | c.(5332+1_5333-1)_(5406+1_5407-1)del |
| 52 | BRCA1 | c.3756 3759del |
| 53 | BRCA1 | c.181T>G |
| 54 | BRCA1 | c.5266dup |
| 55 | BRCA1 | c.1687C> T |
| 56 | BRCA1 | c.5266dupC |
| 57 | BRCA1 | c.4185+1G>T |
| 58 | BRCA1 | c.5266dup |
| 59 | BRCA1 | c.5096G>A |
| 60 | BRCA1 | NC_000017 .10:g.(? 41197819) (41203134 41209069)del |
| 61 | BRCA1 | c.4689C>G |
| 62 | BRCA1 | c.5164T>C |
| 63 | BRCA1 | c.5266dup |
| 64 | BRCA1 | c.(4675+1_ 4676-1)_(5277 +1_5278-l)del heterozygot |
| 65 | BRCA1 | c.628C>T |
| 66 | BRCA1 | c.5333-6T>G |
| 67 | BRCA1 | c.2411_2412del |
| 68 | BRCA1 | c.442-7T>A |
| 69 | BRCA1 | c.131G>T |
| 70 | BRCA1 | c.3481_3491del11 |
| 71 | BRCA1 | c.5377A>T |
| 72 | BRCA1 | c.1154G>A |
| 73 | BRCA1 | c.5492del |
| 74 | BRCA1 | NC_000017 .10:g.(? _ 41197697)_(41203134_ 41209069)del |
| 75 | BRCA1 | c.3700_3704del |
| 76 | BRCA1 | c.3018_3021del |
| 77 | BRCA1 | c.68_69delAG |
| 78 | BRCA1 | c.5266dup |
| 79 | BRCA1 | c.1127del |
| 80 | BRCA1 | c.181T>G |
| 81 | BRCA1 | c.3700_3704del |
| 82 | BRCA1 | c.5266dup |
| 83 | BRCA1 | c.2411_2412del |
| 84 | BRCA1 | c.(5332+1_5333-1)_(5406+1_5407-l)del |
| 85 | BRCA1 | c.(4986+1_ 4987-1)_(5074+1_5075-1)del |
| 86 | BRCA1 | c.3481 3491del |
| 87 | BRCA1 | c.1916T>A |
| 88 | BRCA1 | c.5266dup |
| 89 | BRCA1 | c.(4484+1_4485-1)_(5193+1_5194-1)del |
| 90 | BRCA1 | c.4097-2A>G |
| 91 | BRCA2 | c.6591_6592del |
| 92 | BRCA2 | c.4449del |
| 93 | BRCA2 | c.6952C> T |
| 94 | BRCA2 | c.4131_ 4132insTGAGGA heterozygot |
| 95 | BRCA2 | c.1309_1312delAAAG |
| 96 | BRCA2 | c.1296_1297delGA |
| 97 | BRCA2 | c.3637G> T |
| 98 | BRCA2 | c.5682C>G |
| 99 | BRCA2 | c.1813dupA |
| 100 | BRCA2 | c.2806_2809del |
| 101 | BRCA2 | c.1813dupA heterozygot |
| 102 | BRCA2 | c.2206del |
| 103 | BRCA2 | c.9371A>T |
| 104 | BRCA2 | c.3G>A |
| 105 | BRCA2 | c.2808 2811del |
| 106 | BRCA2 | c.1813dupA |
| 107 | BRCA2 | c.5946del |
| 108 | BRCA2 | c.5035dup heterozygot |
| 109 | BRCA2 | c.516+2T>A |
| 110 | BRCA2 | c.9371A>T |
| 111 | BRCA2 | c.9371A>T |
| 112 | BRCA2 | c.1636del |
| 113 | BRCA2 | c.1813dupC |
| 114 | BRCA2 | c.3599 3600del heterozygot |
| 115 | BRCA2 | c.3191C>G |
| 116 | BRCA2 | c.(8953+1_8954-1)_(9117 +1_9118-1)del |
| 117 | BRCA2 | c.9371A> T |
| 118 | BRCA2 | c.7558C>T |
| 119 | BRCA2 | c.3847 3848del |
| 120 | BRCA2 | c.9435_9436del |
| 121 | BRCA2 | c.(8953+1_8954-1)_(9117+1_9118-1)del |
| 122 | BRCA2 | c.5303_5304del |
| 123 | BRCA2 | c.5164 5165del |
| 124 | BRCA2 | c.7617+2T>G |
| 125 | BRCA2 | c.67+1G>T |
| 126 | BRCA2 | c.516+2T>A |
| 127 | BRCA2 | c.7913_7917del |
| 128 | BRCA2 | c.7007G>A |
| 129 | BRCA2 | c.5217_5224del |
| 130 | BRCA2 | c.5616 5620del |
| 131 | BRCA2 | c.1389 1390del |
| 132 | BRCA2 | g.(? 32944539) (32945237 ?)dup. |
| 133 | BRCA2 | c.5217 5223del |
| 134 | BRCA2 | c.2808 2811del |
| 135 | BRCA2 | c.6405 6409del |
| 136 | BRCA2 | c.8695C> T |
| 137 | BRCA2 | c.1813dup |
| 138 | BRCA2 | c.1813delA |
| 139 | BRCA2 | c. 7863T>A |
| 140 | BRCA2 | c.9257-1G>C |
| 141 | BRCA2 | c.1813del |
| 142 | BRCA2 | c.1813dupA |
| 143 | BRCA2 | c. 7913_7917del |
| 144 | BRCA2 | c.7558C>T |
| 145 | BRCA2 | c.1813dup |
| 146 | BRCA2 | c.3500_3501del |
| 147 | BRCA2 | c.1813dup |
| 148 | BRCA2 | c.1813dup |

Supplementary Table 2 Patient and tumor characteristics with RRSO, showing mean and standard deviation or frequency and percentage for g*BRCA1*mut/g*BRCA2*mut.

|  |  | **Patients with RRSO**  **(n = 101)** | **g*BRCA1*mut**  **(n=61)** | **g*BRCA2*mut**  **(n=40)** |
| --- | --- | --- | --- | --- |
| Age (years) (n/%) | Mean (SD) | 46.7 (±8.2) | 44.1 (±6.8) | 50.7 (±8.2) |
|  | Median (range) | 44.5 (29-72) | 42 (29-68) | 48 (34-72) |
|  | <35 | 8 (7.9) | 7 (11.5) | 1 (2.5) |
|  | ≥35, <50 | 59 (58.4) | 39 (63.9) | 20 (50.0) |
|  | ≥50, <70 | 31 (30.7) | 15 (24.6) | 16 (40.0) |
|  | ≥70 | 3 (3.0) | 0 | 3 (7.5) |
|  | No information available | 0 | 0 | 0 |
| Addtional HGSC (n/%) | Yes | 3 (2.9) | 2 (3.3) | 1 (2.5) |
|  | - Before BC | 0 | 0 | 0 |
|  | - Synchronous | 0 | 0 | 0 |
|  | - After BC | 3 (100) | 2 (100) | 1 (100) |
|  | No | 98 (97.1) | 59 (96.7) | 39 (97.5) |
| BC Relapse (n/%) | Yes | 16 (15.8) | 8 (13.1) | 8 (20.0) |
|  | - Local | 7 (43.8) | 4 (50.0) | 3 (37.5) |
|  | - Local + Advanced | 1 (6.2) | 1 (12.5) | 0 |
|  | - Advanced | 8 (50.0) | 3 (37.5) | 5 (62.5) |
|  | De novo mBC | 4 (4.0) | 1 (1.6) | 3 (7.5) |
|  | No | 81 (80.2) | 52 (85.3) | 29 (72.5) |
| Time to relapse (months) (n/%) | ≤18 | 1 (6.3) | 1 (12.5) | 0 |
|  | >18, ≤36 | 4 (25.0) | 2 (25.0) | 2 (25.0) |
|  | >36, ≤60 | 5 (31.2) | 3 (37.5) | 2 (25.0) |
|  | >60 | 6 (37.5) | 2 (25.0) | 4 (50.0) |
| UICC Stage (n/%) | 0 (DCIS) | 1 (1.0) | 0 | 1 (2.5) |
|  | I | 35 (34.6) | 18 (29.5) | 17 (42.5) |
|  | II | 45 (44.5) | 31 (50.8) | 14 (35.0) |
|  | III | 4 (4.0) | 1 (1.6) | 3 (7.5) |
|  | IV | 2 (2.0) | 1 (1.6) | 1 (2.5) |
|  | No information available | 14 (13.9) | 10 (16.4) | 4 (10.0) |
| BC subtype (n/%) | HR pos | 36 (36.0) | 14 (22.9) | 22 (56.4) |
|  | HER2 pos | 10 (10.0) | 4 (6.6) | 6 (15.4) |
|  | TNBC | 52 (52.0) | 41 (67.2) | 11 (28.2) |
|  | No informations | 2 (2.0) | 2 (3.3) | 0 |

**Supplementary Table 3** Patient and tumor characteristics without RRSO, showing mean and standard deviation or frequency and percentage g*BRCA1*mut/g*BRCA2*mut.

|  |  | **Patients without RRSO**  **(n = 47)** | **g*BRCA1*mut**  **(n = 29)** | **g*BRCA2*mut**  **(n = 18)** |
| --- | --- | --- | --- | --- |
| Age (years) (n/%) | Mean (SD) | 48.2 (±16.2) | 48.6 (±14.1) | 47.5 (±15.0) |
|  | Median (range) | 45 (21-82) | 45.5 (22-82) | 43 (21-79) |
|  | <35 | 12 (25.5) | 8 (27.6) | 4 (22.2) |
|  | ≥35, <50 | 12 (25.5) | 7 (24.1) | 5 (27.8) |
|  | ≥50, <70 | 15 (31.9) | 10 (34.5) | 5 (27.8) |
|  | ≥70 | 6 (12.8) | 3 (10.3) | 3 (16.7) |
|  | No information available | 2 (4.3) | 1 (3.5) | 1 (5.5) |
| Additional HGSC (n/%) | Yes | 7 (14.9) | 4 (13.8) | 3 (16.7) |
|  | - Before BC | 2 (28.6) | 2 (50.0) | 0 |
|  | - Synchronous | 1 (14.2) | 1 (25.0) | 0 |
|  | - After BC | 4 (57.2) | 1 (25.0) | 3 (100) |
|  | No | 40 (85.1) | 25 (86.2) | 15 (83.3) |
| BC Relapse (n/%) | Yes | 11 (23.4) | 6 (20.7) | 5 (27.8) |
|  | - Local | 4 (36.3) | 3 (50.0) | 1 (20.0) |
|  | - Local + Advanced | 2 (18.2) | 0 | 2 (40.0) |
|  | - Advanced | 5 (45.5) | 3 (50.0) | 2 (40.0) |
|  | De novo mBC | 5 (10.6) | 3 (10.3) | 2 (11.1) |
|  | No | 31 (66.0) | 20 (69.0) | 11 (61.1) |
| Time to relapse (months) (n/%) | ≤18 | 2 (18.2) | 2 (33.3) | 0 |
|  | >18, ≤36 | 6 (54.5) | 3 (50.0) | 3 (60.0) |
|  | >36, ≤60 | 1 (9.1) | 1 (16.7) | 0 |
|  | >60 | 2 (18.2) | 0 | 2 (40.0) |
| UICC Stage (n/%) | 0 (DCIS) | 1 (2.2) | 0 | 1 (5.6) |
|  | I | 9 (19.1) | 5 (17.2) | 4 (22.2) |
|  | II | 20 (42.5) | 14 (48.3) | 6 (33.3) |
|  | III | 4 (8.5) | 3 (10.3) | 1 (5.6) |
|  | IV | 6 (12.8) | 5 (17.2) | 1 (5.6) |
|  | No information available | 7 (14.9) | 2 (6.9) | 5 (27.8) |
| BC subtype (n/%) | HR pos | 20 (41.3) | 8 (27.6) | 12 (70.6) |
|  | HER2 pos | 3 (8.5) | 3 (10.3) | 0 |
|  | TNBC | 18 (36.2) | 16 (55.2) | 2 (11.8) |
|  | No informations | 5 (13.0) | 2 (6.9) | 3 (17.6) |

**Supplementary Table 4** BC risk-reducing operations in *gBRCA1*mut and *gBRCA2*mut BC patients with RRSO.

|  |  | **Patients with RRSO**  **(n = 101)** | **g*BRCA1*mut**  **(n=61)** | **g*BRCA2*mut**  **(n=40)** |
| --- | --- | --- | --- | --- |
| Breast Operation (n/%) | Breast conserving therapy | 25 (24.8) | 14 (23.0) | 11 (27.5) |
|  | Mastectomy | 73 (72.3) | 46 (75.4) | 27 (67.5) |
|  | No information available | 3 (2.9) | 1 (1.6) | 2 (5.0) |
| Reconstruction (n/%) | Total | 73 | 46 | 27 |
|  | Subcutaneous Implants | 15 (20.5) | 9 (19.6) | 6 (22.2) |
|  | Subpectoral Implants | 23 (31.5) | 18 (39.1) | 5 (18.5) |
|  | No implants | 35 (48.0) | 19 (41.3) | 16 (59.3) |
| Time to RRSO (months) (n/%) | ≤12 | 52 (51.5) | 30 (49.2) | 22 (55.0) |
|  | >12 - ≤24 | 23 (22.8) | 13 (21.3) | 10 (25.0) |
|  | >24 - ≤60 | 9 (8.9) | 6 (9.8) | 3 (7.5) |
|  | >60 | 16 (15.8) | 12 (19.7) | 4 (10.0) |
|  | Before BC | 1 (1.0) | 0 | 1 (2.5) |

**Supplementary Table 5** BC risk-reducing operations in *gBRCA1*mut and *gBRCA2*mut BC patients without RRSO.

|  |  | **Patients without RRSO**  **(n = 47)** | **g*BRCA1*mut**  **(n = 29)** | **g*BRCA2*mut**  **(n = 18)** |
| --- | --- | --- | --- | --- |
| Breast Operation (n/%) | Breast conserving therapy | 12 (25.5) | 5 (17.2) | 7 (38.9) |
|  | Mastectomy | 25 (53.2) | 18 (62.1) | 7 (38.9) |
|  | No information available | 10 (21.3) | 6 (20.7) | 4 (22.2) |
| Reconstruction (n/%) | Total | 25 | 18 | 7 |
|  | Subcutaneous Implants | 6 (24.0) | 5 (27.8) | 1 (14.3) |
|  | Subpectoral Implants | 8 (21.0) | 5 (27.8) | 3 (42.9) |
|  | No implants | 11 (55.0) | 8 (44.4) | 3 (42.9) |

**Supplementary Table 6** Median time to RRSO in months and RRSO-free probability in *gBRCA*-mutated BC patients. *CI* confidence interval.

| **Records** | **Events** | **Median time to RRSO in months (95% CI)** | **6-month RRSO-free probability (95% CI)** | **12-month RRSO-free probability (95% CI)** | **24-month RRSO-free probability (95% CI)** | **36-month RRSO-free probability (95% CI)** | **60-month RRSO-free probability (95% CI)** | **120-month RRSO-free probability (95% CI)** | **180-month RRSO-free probability (95% CI)** | **240-month RRSO-free probability (95% CI)** |
| --- | --- | --- | --- | --- | --- | --- | --- | --- | --- | --- |
| 125 | 97 | 16.0 (13.0, 24.0) | 0.93 (0.88, 0.97) | 0.58 (0.50, 0.68) | 0.39 (0.31, 0.49) | 0.36 (0.29, 0.46) | 0.30 (0.23, 0.40) | 0.23 (0.16, 0.33) | 0.16 (0.09, 0.27) | 0.06 (0.02, 0.17) |

**Supplementary Table 7** Median time to RRSO in months and RRSO-free probability with respect to guideline recommendation for RRSO in *gBRCA*-mutated BC patients. *CI* confidence interval.

| **Group** | **Records** | **Events** | **Median time to RRSO in months (95% CI)** | **6-month RRSO-free probability (95% CI)** | **12-month RRSO-free probability (95% CI)** | **24-month RRSO-free probability (95% CI)** | **36-month RRSO-free probability (95% CI)** | **60-month RRSO-free probability (95% CI)** | **120-month RRSO-free probability (95% CI)** | **180-month RRSO-free probability (95% CI)** | **240-month RRSO-free probability (95% CI)** |
| --- | --- | --- | --- | --- | --- | --- | --- | --- | --- | --- | --- |
| not (yet) recommended | 24 | 12 | 42.0 (18.0, NA) | 1.00 (1.00, 1.00) | 0.87 (0.74, 1.00) | 0.59 (0.42, 0.84) | 0.53 (0.35, 0.80) | 0.46 (0.28, 0.75) | 0.46 (0.28, 0.75) | 0.23 (0.05, 1.00) | 0.23 (0.05, 1.00) |
| recommended | 101 | 85 | 15.0 (11.0, 19.0) | 0.91 (0.86, 0.97) | 0.52 (0.43, 0.63) | 0.34 (0.26, 0.45) | 0.32 (0.24, 0.43) | 0.26 (0.19, 0.37) | 0.19 (0.12, 0.30) | 0.14 (0.08, 0.25) | 0.04 (0.01, 0.15) |

**Supplementary Table 8** STROBE Statement—checklist of items that should be included in reports of observational studies

|  | Item No. | Recommendation | Page  No. | Relevant text from manuscript |
| --- | --- | --- | --- | --- |
| **Title and abstract** | 1 | (*a*) Indicate the study’s design with a commonly used term in the title or the abstract | 1 | Risk reducing Salpingo-Oophorectomy in Patients with gBRCA-mutated Breast Cancer |
|  |  | (*b*) Provide in the abstract an informative and balanced summary of what was done and what was found | 2 | This retrospective study included 148 patients with gBRCA1/2mut BC treated at the Hereditary Breast and Ovarian Cancer Center, University Hospital Erlangen, between 2012 and 2024. Clinical, pathological, treatment-related, and genetic data were collected and analyzed from electronic records. Time to operate RRSO at date of BC surgery was calculated for all patients, and stratified according guideline recommendations for RRSO, using the Kaplan-Meier method. |
| Introduction | | | |  |
| Background/rationale | 2 | Explain the scientific background and rationale for the investigation being reported | 5 | Previous studies, including those by Blondeaux et al. and Willert et al., have demonstrated that RRSO is associated with a significant reduction in OC incidence, while the uptake of risk-reducing surgery has increased substantially over recent years. Nevertheless, evidence defining the optimal timing of RRSO remains limited. Current national and international guidelines recommend offering RRSO from the age of 35 years in BRCA1 and 40 years in BRCA2 pathogenic variant carriers. However, they do not provide specific recommendations regarding the timing of surgery in relation to BC treatment. |
| Objectives | 3 | State specific objectives, including any prespecified hypotheses | 5 | The primary objective of this study was to evaluate the timing of RRSO in patients with gBRCAmut BC and to determine the interval between BC diagnosis and RRSO in a real-world setting. In addition, the study aims to characterize the patient cohorts, and to assess the incidence OC in RSSO versus the occurrence of OC in non-operated gBRCAmut BC patients during follow-up. |
| Methods | | | |  |
| Study design | 4 | Present key elements of study design early in the paper | 6,7 | Between January 1, 2012, and December 31, 2024, 3,599 individuals presented at the HBOC Center at the University Hospital Erlangen.  Medical histories and results of human genetic testing were documented in an electronic case report form using Microsoft Access 365 |
| Setting | 5 | Describe the setting, locations, and relevant dates, including periods of recruitment, exposure, follow-up, and data collection | 6,7 | Patients eligible for genetic counseling are identified through a systematic assessment of the German Cancer Society's inclusion criteria. If the criteria are met, patients can undergo counselling at the HBOC center  Follow-up assessments for the BC setting are updated every 6 months until 5 years after BC diagnosis. An intensified breast cancer surveillance is recommened for gBRCAmut BC patients without a mastectomy.  . |
| Participants | 6 | (*a*) *Cohort study*—Give the eligibility criteria, and the sources and methods of selection of participants. Describe methods of follow-up.  *Case-control study*—Give the eligibility criteria, and the sources and methods of case ascertainment and control selection. Give the rationale for the choice of cases and controls  *Cross-sectional study*—Give the eligibility criteria, and the sources and methods of selection of participants | 6, 8, 9 | 3,599 patients were registered in the database. Of these, non-affected individuals (n = 1,050), affected patients with non-BC (n = 383), and patients without a gBRCA mutation (n = 1,954)…..  Of 148 patients, 21 patients were excluded due to missing operation-free time in months. Subsequently, 2 patients….. |
|  |  | (*b*) *Cohort study*—For matched studies, give matching criteria and number of exposed and unexposed  *Case-control study*—For matched studies, give matching criteria and the number of controls per case | NA | NA |
| Variables | 7 | Clearly define all outcomes, exposures, predictors, potential confounders, and effect modifiers. Give diagnostic criteria, if applicable | 5,8 | The primary objective of this study was to evaluate the timing of RRSO in patients with gBRCAmut BC and to determine the interval between BC operation and RRSO in a real-world setting.  Operation-free survival was defined from the date of BC operation to the date of RRSO or the last operation-free date known. Operation-free last date known was date of death, if death happened without operation…. |
| Data sources/ measurement | 8* | For each variable of interest, give sources of data and details of methods of assessment (measurement). Describe comparability of assessment methods if there is more than one group | 7 | Patient data from the period between January 1, 2012, and December 31, 2024, at the HBOC Center at the University Hospital Erlangen, were collected retrospectively by trained personnel. Medical histories and results of human genetic testing were documented in an electronic case report.  Plausibility checks issued regarding the research question, and on-site monitoring was performed. |
| Bias | 9 | Describe any efforts to address potential sources of bias | 7 | Ascertainment bias likely resulted in and under-reporting in our real-world trial similar to other real-world studies. |
| Study size | 10 | Explain how the study size was arrived at | 7 | 3,599 patients were registered in the database. …... Ultimately, 148 patients with gBRCAmut BC/DCIS were included in this analysis. |

Continued on next page

| Quantitative variables | 11 | Explain how quantitative variables were handled in the analyses. If applicable, describe which groupings were chosen and why | 7,8 | Patient and tumor characteristics were described using summary statistics. Frequency and percentage were used for categorical variables.  Survival rates with 95% confidence intervals (CIs) and median survival times were estimated using the Kaplan-Meier product limit method. The 95% CI of median survival times were computed using the method of Brookmeyer and Crowley. |
| --- | --- | --- | --- | --- |
| Statistical methods | 12 | (*a*) Describe all statistical methods, including those used to control for confounding | 7,8 | Operation-free survival was defined from the date of BC operation to the date of RRSO or the last operation-free date known.  … For patients in group A, RRSO was recommended, for patients in group B it was not (yet) recommended |
|  |  | (*b*) Describe any methods used to examine subgroups and interactions | 21 | Subgroup analyses were conducted based on the following variables: age (categorical; up to 35 years, 35 to 50 years, etc), BRCA mutation, metachronous HGSC, BC relapse.… |
|  |  | (*c*) Explain how missing data were addressed | 6,8 | 3,599 patients were registered in the database. Of these, non-affected individuals (n = 1,050), affected patients with non-BC (n = 383), and patients without a gBRCA mutation (n = 1,954) were excluded. A gBRCA mutation was detected in 212 patients with BC/DCIS. From this population, data were unavailable for an additional 61 patients, mainly treated in other certified BC centers. As HBOC testing is restricted to selected centers, these patients presented in this clinic just for genetic counseling and testing. 3 male patients were excluded furthermore  Of 148 patients, 21 patients were excluded due to missing operation-free time in months. Subsequently, 2 patients had to be excluded due to non-positive survival time. Additionally, patients were excluded if germline testing was performed only after the diagnosis of mBC for therapeutic decision-making. Patients were also excluded if they developed OC during BC follow-up and consequently underwent salpingo-oophorectomy, or if no further clinical information was available because surgery had been performed at an external institution. Therefore, 125 patients were eligible for analyzing time to operate with survival analysis. |
|  |  | (*d*) *Cohort study*—If applicable, explain how loss to follow-up was addressed  *Case-control study*—If applicable, explain how matching of cases and controls was addressed  *Cross-sectional study*—If applicable, describe analytical methods taking account of sampling strategy | 8 | Operation-free last date known was date of death, if death happened without operation. If patient was lost to follow up before death or operation, operation-free last date was date of lost to follow up. Otherwise, operation-free last date known was date of data extraction, which was December 31th, 2024. Operation-free survival was right-censored at 240 months (= 20 years). |
|  |  | (*e*) Describe any sensitivity analyses | NA | NA |
| Results | | | | |
| Participants | 13* | (a) Report numbers of individuals at each stage of study—eg numbers potentially eligible, examined for eligibility, confirmed eligible, included in the study, completing follow-up, and analysed | 6 | At the time of data cut-off (December 31, 2024), 3,599 patients were registered in the database. Of these, non-affected individuals (n = 1,050), affected patients with non-BC (n = 383), and patients without a gBRCA mutation (n = 1,954) were excluded. A gBRCA mutation was detected in 212 patients with BC/DCIS. From this population, data were unavailable for an additional 61 patients, mainly treated in other certified BC centers. As HBOC testing is restricted to selected centers, these patients presented in this clinic just for genetic counseling and testing. 3 male patients were excluded furthermore. Ultimately, 148 patients with gBRCAmut BC/DCIS were included in this analysis. |
|  |  | (b) Give reasons for non-participation at each stage | 6 | Non-affected individuals (*n* = 1,050), affected patients with non-BC (*n* = 383), and patients without a *g*BRCA mutation (*n* = 1,954) were excluded. … From this population, data were unavailable for an additional 61 patients, mainly treated in other certified BC centers. 3 male patients were excluded furthermore. Ultimately, 148 patients with *g*BRCA*mut* BC/DCIS were included in this analysis. |
|  |  | (c) Consider use of a flow diagram | 17 | Figure 1 |
| Descriptive data | 14* | (a) Give characteristics of study participants (eg demographic, clinical, social) and information on exposures and potential confounders | 8, 21 | Baseline patient and tumor characteristics are shown in Table 1. |
|  |  | (b) Indicate number of participants with missing data for each variable of interest | 8, 21 | Baseline patient and tumor characteristics are shown in Table 1. |
|  |  | (c) *Cohort study*—Summarise follow-up time (eg, average and total amount) | 10 | Median operation-free observation time in months with interquartile range was 16.00 (10.00, 51.00). |
| Outcome data | 15* | *Cohort study*—Report numbers of outcome events or summary measures over time | 19, 20 | Events for time to operate RRSO are given in Tab 2 und Tab 3; Median survival time is given in Supplement Tab 6 and Tab 7 |
|  |  | *Case-control study—*Report numbers in each exposure category, or summary measures of exposure | NA | NA |
|  |  | *Cross-sectional study—*Report numbers of outcome events or summary measures | NA | NA |
| Main results | 16 | (*a*) Give unadjusted estimates and, if applicable, confounder-adjusted estimates and their precision (eg, 95% confidence interval). Make clear which confounders were adjusted for and why they were included | 9 | Median operation-free observation time in months with interquartile range was 16.00 (10.00, 51.00).  No confounder-adjustement was carried out. |
|  |  | (*b*) Report category boundaries when continuous variables were categorized | 21 | For example for age: up to 35 years, 35 to 49 years, 50 to 69 years, 70 years or older…. |
|  |  | (*c*) If relevant, consider translating estimates of relative risk into absolute risk for a meaningful time period | NA | NA |

Continued on next page

| Other analyses | 17 | Report other analyses done—eg analyses of subgroups and interactions, and sensitivity analyses | Supp. 7 | Further Median RRSO-free survival times and rates are shown in Supplemental Tab 6 and 7 for 6, 12, 24, etc months |
| --- | --- | --- | --- | --- |
| Discussion | | | | |
| Key results | 18 | Summarise key results with reference to study objectives | 12 | In the single-center retrospective analysis of patients with gBRCAmut BC, most patients underwent RRSO, predominantly within 12 months after BC diagnosis. Occult OC was detected in 3 out of 101 patients at time of RRSO, whereas 4 out of 47 of patients not undergoing RRSO developed OC after their BC diagnosis. |
| Limitations | 19 | Discuss limitations of the study, taking into account sources of potential bias or imprecision. Discuss both direction and magnitude of any potential bias | 13 | Our study has several limitations. The retrospective design leads to the introduction of several biases, selection bias cannot be excluded, as patients who underwent RRSO may differ systematically from those who did not. Missing or incomplete data, differing follow-up times and other confounding factors may have affected the outcome assessment. Owing to the single-center design, the cohort size is limited. The relatively small cohort size precluded meaningful inferential statistical analyses. Therefore, the data were analyzed descriptively. Given the limited sample size, the risk of statistical error was considered substantial. In addition, the single-center design may have introduced selection bias related to local clinical counseling and referral practices. Nevertheless, genetic counseling and clinical decision-making were conducted in accordance with current national and international guidelines. There is a further limitation for interpretations as data were collected in Germany only. There, transvaginal ultrasonography as an early detection is not recommended. Only risk-reducing surgery is reimbursed by health insurance providers. Owing to the limited evidence base, no guideline recommendation for transvaginal ultrasonography currently exists in Germany, in contrast to some other countries. This more directive approach to genetic counselling should be taken into account when interpreting the results. |
| Interpretation | 20 | Give a cautious overall interpretation of results considering objectives, limitations, multiplicity of analyses, results from similar studies, and other relevant evidence | 14 | The optimal timing for RRSO in gBRCA1/2 mutation carriers remains challenging. The procedure is generally recommended between the age of 35 and 45 years, depending on the specific mutation. When HBOC is identified due to BC diagnosis, more than 50% of patients opt to undergo RRSO within 12 months. The rate of occult carcinomas appears to be low. Nevertheless, given the continuous improvements in BC treatment and the associated increase in OS, the cumulative risk of subsequent malignancies remains clinically relevant over time. Despite a prior BC diagnosis, RRSO should continue to be discussed with gBRCAmut patients during BC follow-up. These data may provide valuable support during this complex decision-making process and facilitate shared decision-making to determine the most appropriate, individualised timing of RRSO |
| Generalisability | 21 | Discuss the generalisability (external validity) of the study results | 12,13 | Two-thirds of patients underwent RRSO, more than 50% undergoing surgery within the first 12 months of BC diagnosis, in total 75% within two years. 16% of the patients underwent RRSO more than five years after BC diagnosis. Especially in patients with BC before the age of 35 RRSO was often performed later but still within the internationally recommended timeframe for risk-reducing surgery. …..  ….. In our cohort, occult ovarian carcinoma was likewise identified in nearly 3% of patients undergoing RRSO: two cases among 61 g*BRCA1* mutation carriers and one case among 40 *gBRCA2* mutation carriers. Although the sample size is very limited, there could be a tendency towards a higher prevalence of occult carcinoma in g*BRCA1* mutation carriers, consistent with the findings of Finch et al., who reported prevalences of 4.2% for g*BRCA1* and 0.6% for g*BRCA2* mutation carriers. …….  In the context of precision oncology, OS in BC patients is improving. It may therefore be assumed that the risk of second primary malignancies could also increase in gBRCAmut patients during BC follow-up. Apostol et al. have demonstrated an ongoing OC in gBRCAmut BC patients, with a pronounced high risk in gBRCA1 mutations carriers. Accordingly, RRSO should continue to be recommended for gBRCAmut BC patients as a preventive strategy. |
| Other information | |  | | |
| Funding | 22 | Give the source of funding and the role of the funders for the present study and, if applicable, for the original study on which the present article is based | 15 | This research received no external funding. |

*Give information separately for cases and controls in case-control studies and, if applicable, for exposed and unexposed groups in cohort and cross-sectional studies.

**Note:** An Explanation and Elaboration article discusses each checklist item and gives methodological background and published examples of transparent reporting. The STROBE checklist is best used in conjunction with this article (freely available on the Web sites of PLoS Medicine at http://www.plosmedicine.org/, Annals of Internal Medicine at http://www.annals.org/, and Epidemiology at http://www.epidem.com/). Information on the STROBE Initiative is available at www.strobe-statement.org.
